# Cancer incidence in the middle region of Libya: Data from the cancer epidemiology study in Misurata

**DOI:** 10.1002/cnr2.1448

**Published:** 2021-06-15

**Authors:** Anas Zarmouh, Abdulrahman Almalti, Ahmad Alzedam, Marwa Hamad, Hamad Elmughrabi, Laila Alnajjar, Mohammed Elmugassabi, Muataz Kashbour, Wesam Elsaghayer

**Affiliations:** ^1^ Department of Medicine, Faculty of Medicine University of Misurata Misrata Libya; ^2^ Misurata Medical Center Misrata Libya; ^3^ National Cancer Institute Misrata Libya; ^4^ Tripoli Central Hospital Tripoli Libya; ^5^ Faculty of Medicine University of Misurata Misrata Libya

**Keywords:** cancer, epidemiology, incidence, registry

## Abstract

**Background:**

Cancer incidence and cancer registries are essential for local epidemiological information. In Libya, scarce evidence exists with regard to incidence rates and distribution.

**Aim:**

To estimate cancer incidence in Libya and draw trends of cancer type distribution compared to regional and worldwide data. Such incidence data are needed to inform strategic decisions on cancer facilities, training, and research in the given geographical area of Misurata, the major city in the middle region and third largest in Libya.

**Methods:**

This is an observational, multi‐centre, city‐wide study to account for all cancer cases. All radiology (computed tomography and magnetic resonance imaging) and pathology reports were examined across all public and private hospitals in and around Misurata.

**Results:**

Four hundred and thirty cancer cases were identified to have been diagnosed during 12 months (July 2019–June 2020), yielding a cancer incidence of 71.7 per 100 000 population. Breast cancer (84, 19.5%), colorectal cancer (83, 19.3%), lung cancer (33, 7.7%), and prostate cancer (21, 4.9%) had the highest prevalence.

**Conclusion:**

Cancer incidence established in this study stands at 71.1, much lower than the worldwide reported incidence of 201.0. Several limitations lead to missing cancer cases from the survey period, mostly related to poor documentation, non‐research friendly environment, and disorganised healthcare structure. Nevertheless, distribution by type represents a true contrast to the world cancer report. Finally, a national or regional inclusive cancer registry is essential to the flow of information that supports strategic planning and decision‐making in developing cancer care in the country.

## 1 INTRODUCTION

Cancer is an important cause of morbidity and mortality worldwide. It is the first or second leading cause of premature death in 134 of 183 countries.^1^ The predicted global cancer burden is expected to exceed 27 million new cancer cases per year by 2040. Such an increase will be most significant in underdeveloped countries.^1^

According to the WHO world cancer report 2020, which estimated cancer incidence at 19.3 million new cases, the six most common cancer types worldwide are lung, breast, colorectal, prostate, stomach, and cervical cancer.^1^ Libya has no reliable contribution to this representative data, as there is no comprehensive national or local cancer registry to reliably account for the numbers, types, morbidity, and mortality of cancer.

Cancer registry in Arab countries: countries reporting in IARC volume 9 of CI5 (cancer in 5 continents) series are Kuwait, Oman, Algeria, Bahrain, Egypt, and Tunisia. Regarding other Arab countries, cancer incidence data are found on the Globocan 2020 website.^2^

In male population, lung cancer is the most frequent neoplasm in all the Arab world, except for Yemen, Saudi Arabia, and Mauritania, in which the most common cancers are oral, liver, and prostate, respectively. Breast cancer, almost without exception, is the most frequent tumour type in females.^3^ Liver and bladder cancers are exceptionally predominant in Egypt.^4^

In Libya, local hospital‐based cancer registries declare total numbers diagnosed and treated without any geographical reference, making it impossible to arrive at a cancer incidence rate in relation to the population of the area covered by the hospital.

The literature has scattered articles about cancer epidemiology in Libya. In 2017, a report accounted for 1051 new cancers seen in Tripoli Medical Centre, but the study had no geographical limits as it is a regional centre.^5^ Similarly, 1160 were registered in Benghazi Medical Centre in Libya in 2003 but still lacked external validity.^6^ Both published registers did not attempt to account for all cancers, whether seen in the medical centre or otherwise, that is, cancers were included even if they were outside Benghazi or Tripoli.

Knowledge of cancer epidemiology contributes not only to the national census but also serves to guide strategic health planning, structuring, capacity building, staff recruitment, and even medical research, education, and training.

By default, setting up and maintaining a national cancer registry is a central duty of the ministry of health and a recommendation by the WHO and IARC.^1^, ^7^ However, no such setup exists, and no ongoing local registry produces data relevant to a specific geographical population.

The research team came together to take the initiative to gather diagnostic information to estimate the incidence of cancer in the geographical location of Misurata, which is the largest city in the middle region and third largest in Libya, as well as clarifying other epidemiological features such as age and type distribution compared with previous regional studies in Eastern Libya and the rest of the world.

## 2 METHODS

### 2.1 Study type

The project is an observational, prospective, multi‐centre city‐wide study (survey) to calculate the annual incidence of cancer in Misurata, Libya. The survey lasted for 12 months between July 7, 2019 and June 30, 2020.

### 2.2 Study setting

Sixteen centres in and around Misurata were identified; these include the National Cancer Institute (NCI), a dedicated national cancer centre, Misurata Medical Centre (MMC), a tertiary regional centre, and 14 private hospitals.

#### 2.2.1 Inclusion and exclusion criteria

- All public and private healthcare facilities were included in the study.
- All patients who have had cross‐sectional imaging, that is, computed tomography (CT) or magnetic resonance imaging (MRI), or pathological sample, were included in the survey.
- Reports with a malignant histopathology diagnosis were included.
- Non‐malignant histopathology reports were excluded.
- Imaging reports highly suggestive of malignancy were included but after considerations by a senior member of the team.
- Imaging with non‐malignant reports was excluded.

#### 2.2.2 Data collection procedure

Pathology and imaging reports were collected directly from the clinical team, regularly (weekly to monthly depending on work size) by the research team, filtered to exclude non‐malignant reports. The research team only examined the reports rather than the images or samples. Patients/relatives were contacted to confirm or refute the diagnosis. Towards the end of the project, imaging reports and pathology reports for the single patient were married to account for a single case of cancer in the database. Patients who have not had a confirmatory pathological diagnosis of cancer were phoned by the research team to ask if and when and where a cancer diagnosis has been confirmed or excluded. Missing information was discussed within the research team, and a decision is made to include or exclude it as a cancer case. Cancers were represented according to the WHO world cancer report,^1^ and Globocan 2020 report,^7^ that is, data on lung cancer were not represented in its subtypes, but only as lung cancer. There have been several meetings and workshops to train the newly graduated doctors in the research team on examining and filtering reports. Finally, coding and classification followed the WHO world cancer report and Globocan 2020 report.^8^

To ensure no overestimation of cancer, cases were dismissed if the city was not confirmed, or cancer was not confirmed new for the study period, or CT/MRI reported a possibility, but no histopathological confirmation was found.

In addition to the above data collection procedure being carried out at the NCI, other cases were collected from the institute's own registry, provided the address of Misurata was documented.

All cancer cases were recorded on a data spreadsheet. Further processing, including filtration, sorting, matching, and final analysis, was conducted using the same spreadsheet.

## 3 RESULTS

Sixteen centres had all their radiology and pathology surveyed and filtered for a diagnosis of cancer. Initial readings survey resulted in 874 rows on the collection sheet, marrying names (e.g., CT and pathology report of the same person) and dismissing reports of no cancer and reports with no specific city address resulted in a final number of 430 cases of cancer over 12 months in Misurata.

**FIGURE 1:**
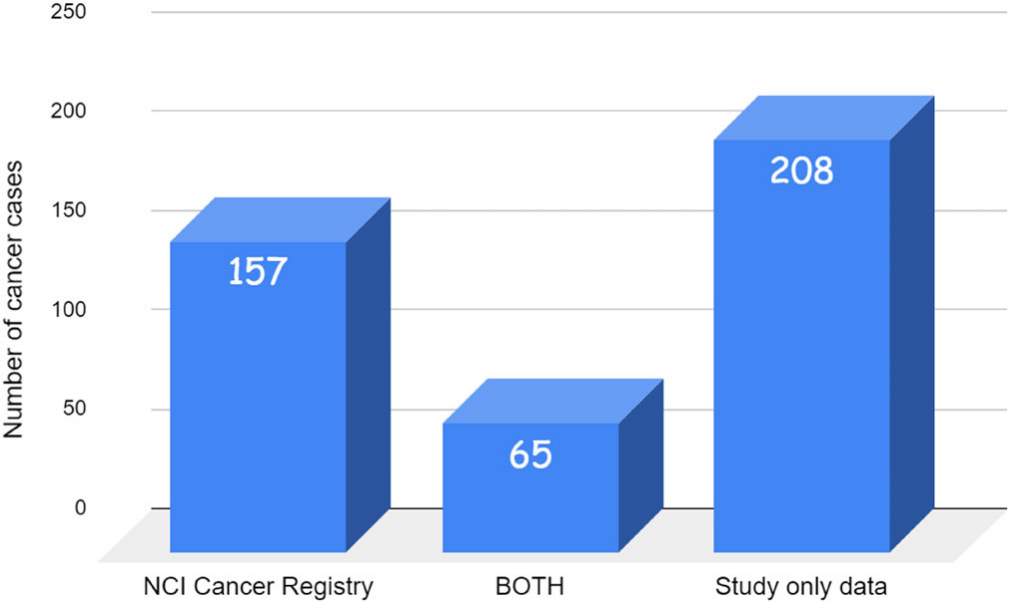
Cancers identified by the study only and those found in the NCI's registry and cancers that were both present in the study and NCI registry

Of the 430 cancer cases, 208 (48.4%) were identified by the current study but were not found in the NCI's cancer registry; 157 (36.5%) appeared only in the NCI's cancer registry but were missed by the study. Only 65 (15.1%) were covered by both the current study and NCI's cancer register (Figure 1).

**FIGURE 2:**
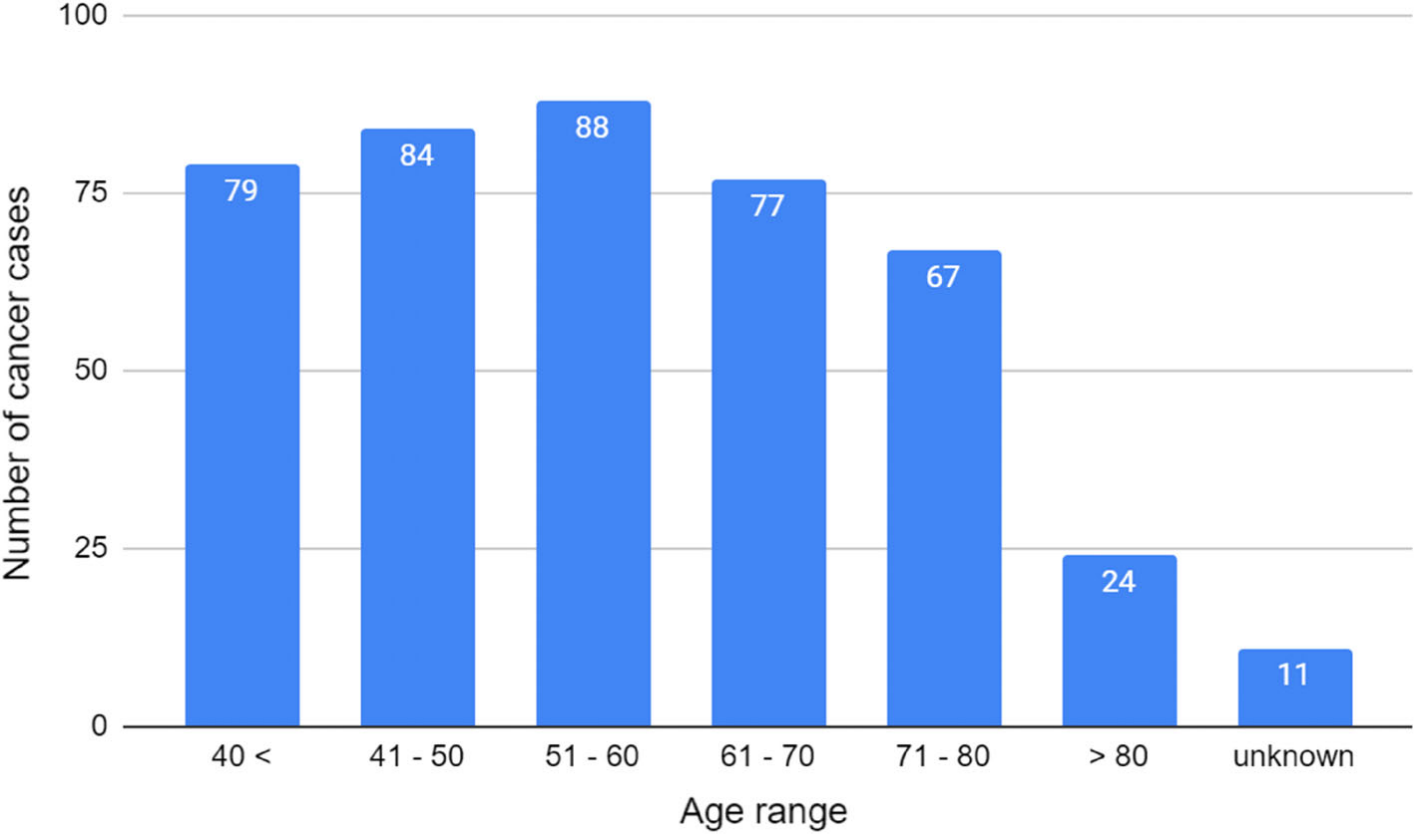
Age distribution of all cancers identified in the survey

There were 192 (44.7%) males with cancer and 238 (55.3%) females with cancer, giving a ratio of 1:1.24. Figure 2 shows the age distribution of cancer cases demonstrates the sixth decade having the highest number of cancers at 88 (20.5%), followed by the fifth decade at 84 (19.5%), as in Figure 2.

**FIGURE 3:**
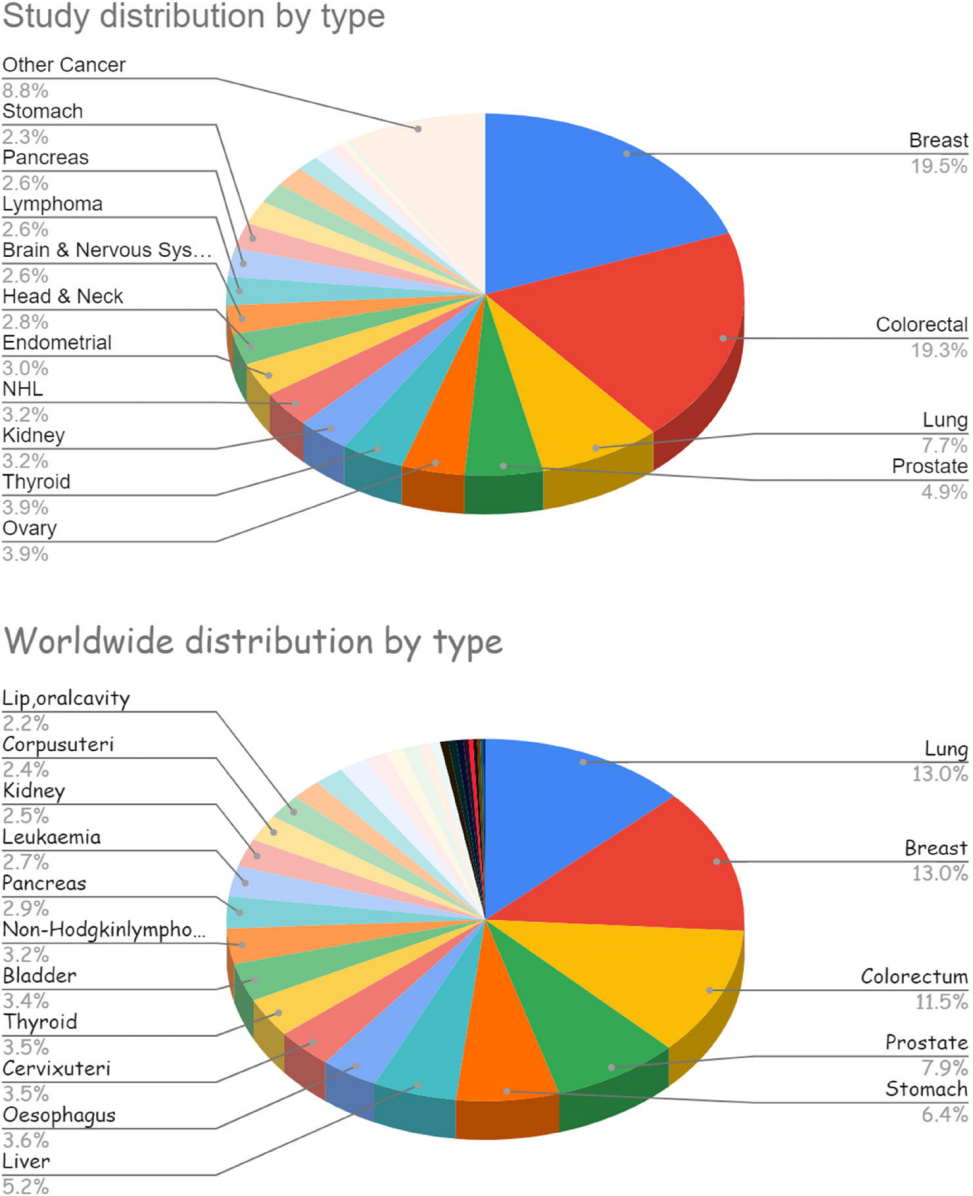
Study and worldwide distribution by type of cancer

Table 1 and Figure 3 demonstrate cancer distribution by type, as contrasted to globocan report and world cancer report 2020. Notably, breast cancer (84, 19.5%), colorectal cancer (83, 19.3%), lung cancer (33, 7.7%), and prostate cancer (21, 4.9%) had the highest prevalence in the study, as compared to lung (13.0%), breast (13.0%), colon (11%), and prostate (7.9%) in the world cancer report.

**TABLE 1:** Study and worldwide distribution by type of cancer

Study cancer cases			Worldwide		
All cancers	430	100.0%	All cancers	18 078 957	100.0%
Breast	84	19.5%	Lung	2 093 876	11.6%
Colorectal	83	19.3%	Breast	2 088 849	11.6%
Lung	33	7.7%	Colorectal	1 849 518	10.2%
Prostate	21	4.9%	Prostate	1 276 106	7.1%
Ovary	17	4.0%	Stomach	1 033 701	5.7%
Thyroid	17	4.0%	Liver	841 080	4.7%
Kidney	14	3.3%	Oesophagus	572 034	3.2%
NHL	14	3.3%	Cervix	569 847	3.2%
Endometrial	13	3.0%	Thyroid	567 233	3.1%
Head and neck	12	2.8%	Bladder	549 393	3.0%
Brain and nervous system	11	2.6%	Non‐Hodgkin lymphoma	509 590	2.8%
Lymphoma	11	2.6%	Pancreas	458 918	2.5%
Pancreas	11	2.6%	Leukaemia	437 033	2.4%
Stomach	10	2.3%	Kidney	403 262	2.2%
Bladder	9	2.1%	Endometrial	382 069	2.1%
Leukaemia	8	1.9%	Lip, oral cavity	354 864	2.0%
Skin	8	1.9%	Brain, central nervous system	296 851	1.6%
Liver	6	1.4%	Ovary	295 414	1.6%
Cervix	5	1.2%	Melanoma of skin	287 723	1.6%
Gallbladder	3	0.7%	Gallbladder	219 420	1.2%
Multiple myeloma	2	0.5%	Larynx	177 422	1.0%
Oesophageal	1	0.2%	Multiple myeloma	159 985	0.9%
Other cancer	38	8.8%	Other cancers	2 654 769	14.7%

## 4 DISCUSSION

This cancer epidemiological survey has identified a total of 430 new cancer cases in Misurata, a city with a population of 600 000 over 12 months, giving a cancer incidence rate of 71.7 per 100 000 population. The most frequently encountered cancer being breast cancer with 84, 19.5%. The pattern of cancers identified in the survey does contrast with the world pattern.

This is the first of such surveys in western Libya, the importance of which is to identify the magnitude and patterns of cancer incidence in a given geographical area, allowing for generalizability of results across the country. The study methodology is similar to the Benghazi cancer registry published by El Mistiri, 6 which had a 62.3% per 100 000 population and reported differences in distribution by type compared to the world cancer report. Because of the lack of an accurate cancer registry and the anticipated variations between both studies, it is impossible to draw evidence of a concrete regional difference between Libya's eastern and western regions. A reliable cancer registry does not exist in Libya, primarily due to the long‐term breakdown of government healthcare institutes, corruption, and political instability over the decades.

The other study conducted in Tripoli and published in 2017 reported 1051 cancers during the year 2008, but those were only sourced from a single cancer registry of a large medical centre. The latter study reported cancer distribution by type and age but did not claim incidence in a given population or geographical location.^5^

To ensure the study was limited to the demographic area of Misurata, and to allow no overestimation of cancer cases, and limitation of the study to the demographic area of Misurata, patients with no identified address or city, patients with a doubtful date of diagnosis, and patients with equivocal radiology reporting were all excluded from the study.

According to a SWOT analysis, it is highly anticipated that underestimation is a major feature of the study; these have been categorised into internal/team‐related weaknesses and external/environmental threats. Internal factors, presumably areas to be improved, included (1) lack of cooperation from medical staff to submit reports, for example, in one major centre, hundreds of reports were not submitted merely because of lack of doctors cooperation with the research team, (2) lack of cooperation from reception or nursing staff to register or request a phone number or city name from patients to allow future communication from the research team, and (3) incomplete data from case reports, making the date of diagnosis unclear and therefore dismissal of the case. Despite the research team's best efforts to mitigate these factors, it is clear that the study missed an unaccounted for a number of patients with cancer. On the contrary, it may be argued that due to the extensive nature of the survey sites, missing reports from one centre could appear in another centre where further testing (radiology or histopathology) took place, allowing the cancer case to be identified. This could also apply to patients who are identified not on their first scan but their follow‐up scan or repeat biopsy.

Factors potentially underestimating cancer numbers in the study include: (1) patients who seek medical advice and complete management outside the country, which is relatively common and unaccounted for. Common destinations include Jordan, Tunisia, and Turkey. However, it may be argued that the corona pandemic has hindered the majority of travel in the country, and henceforth, more cancers would have been diagnosed and treated locally. (2) Cancers that are diagnosed through different means other than imaging and pathology (i.e., peripheral blood films, Bence‐Jones proteins, CXR, or laparotomy). (3) Undiagnosed patients due to poor healthcare provisions, health illiteracy, or even lack of access to healthcare. (4) Inaccurate population leading to inaccurate incidence rate, due to lack of official consensus and the substantial, unpredictable demographic changes in a war‐torn country.

A total of 208 (48.4%) cancers were missing from the NCI's own Misurata cancer registry, as identified by the current survey. Reasons here include (1) patients not being referred to NCI by the treating doctors, (2) patients hesitating to attend NCI, (3) patients having all treatments outside the country, and not passing through NCI at any point, but having an initial diagnosis at a private site, and (4) potential innate documentation issues within the NCI's registry. The current study's methodology either fully or partially covered all these factors. Finally, this indicates that relying on the local NCI registry to draw generalisations on the whole city is biased and flawed.

Accordingly, conclusions and future planning and strategic decisions can be based on the study, without the noise of overestimation, but bearing in mind the underestimations the study faced.

In terms of distribution by type, the study demonstrates a significant contrast to the World Cancer Report and the Fact Sheet published by the IARC for 2020.^1^, ^8^ This contrast is arguably realistic, as all the study limitations mentioned above would have impacted the ratios to a similar degree, henceforth, and while absolute cancer incidence per population by type may be biased given the study limitations, the ratios do reflect distribution by type to a large extent.

Lung cancer came only in third place with 7.7% (33 patients), which is a significant contrast to the established most common cancer in the World Cancer Report at 13.0% (41% lesser lung cancers than the rest of the world). The total of 27 male lung cancers out of 192 overall male cancers represents a 14.1% distribution, comparable to 14.3% of the worldwide male cancers in 2020. This is, however, not reflected in El Mistiri's 2003 report of 21.1% lung cancer in males and 2% in females.^6^ Similarly, 15.6% in males and 4.8% in females were reported for Western Libya in 2017.^5^ According to the IARC, Algeria and Tunisia reported 15.2% and 24.4% incidence of lung cancer in males, respectively.^9^ Clinician and patient‐related factors of symptom recognition, under‐diagnosis, under‐recognition, and overseas treatment may contribute to the significant discrepancies here, rather than the initially perceived strong cultural impact of women not smoking, and therefore the low incidence of lung cancer.

Breast cancer came first with 19.5% (84 patients), also significantly higher than the 13.0% quoted for the rest of the world, that is, 33.3% more frequent breast cancers in Misurata. Colorectal cancer was second in the study, with 19.3% (83 patients), significantly higher by 40% than the 11.5% reported worldwide. Algeria and Tunisia reported 21.5% and 15.9% incidence of breast cancer, respectively, and reported 11.2% and 9.6%, respectively, for colorectal cancers.^9^ It is likely that both breast and colorectal cancer rates may have been offset by the potential underestimation of lung cancer rates; for all we know, the country has no screening policies or cancer awareness programmes for breast, colorectal, or otherwise.

The study's prostate cancer was also significantly contrasted with 4.9% (21 patients) compared with 7.9% worldwide, giving 37.9% fewer cancers; 6.2% and 6.1% were reported for both Algeria and Tunisia in the IARC report.^9^

The present study and the world cancer report have the same four top cancers, albeit in different order of distribution, all accounting for 51.4% of all cancer incidence in the present study, but with 40.5% in the world cancer report. Algeria and Tunisia reported the top 4 cancer cumulative incidence at 47.1% and 47.8%, respectively.^9^

Otherwise, the study ranked ovary, thyroid, kidney, non‐Hodgkin's lymphoma, endometrial, and head/neck in the top 10, respectively. The world cancer report ranked stomach, liver, oesophagus, cervix uteri, thyroid, and bladder among the top 10, respectively.

The variabilities and differences in cancer incidence in the study are related to cultural, environmental, genetic, nutritional, and occupational factors, the analysis of which is outside the scope of the study. However, healthcare‐related factors also influence cancer incidence rates as well as distribution. These include (1) The presence of more expertise in one branch could have facilitated more diagnoses than others where clinical expertise is less abundant. (2) The availability of awareness programmes to target specific populations may give rise to more cancers diagnosed than the non‐promoted. (3) Inherent study‐related factors related to under‐estimation and over‐estimation of cancers numbers of the survey.

Furthermore, this survey faced significant hurdles when collecting data, mainly related to the many data collection sites (16 sites) and differing attitudes to epidemiological research amongst healthcare workers (doctors, nurses, and support staff), yielding a non‐research friendly environment. Accordingly, it was not possible to collect data on non‐cancer cases to allow for analysis of excluded cases to account for any bias relating to site, speciality, or private versus public. Future work tackling such extensive but essential horizons should take into account the many burdensome, exhausting, and in some instances, non‐remediable barriers.

## 5 CONCLUSION

In Misurata, cancer incidence stands at 71.7 per 100 000 population with 430 cases during the 12 month survey period (July 2019–June 2020). This incidence rate is at least an underestimate, with differences in distribution by type to the world populations. Furthermore, a single centre cancer registry is an unreliable alternative to a national or regional cancer registry that collects information from all healthcare facilities in the region. All the above should support strategic planning and decision‐making in developing cancer care in the country.^7^

## CONFLICT OF INTEREST

All authors declare no conflict of interest.

## AUTHOR CONTRIBUTIONS

All authors had full access to the data in the study and take responsibility for the integrity of the data and the accuracy of the data analysis. *Conceptualization*, A.Z., A.A., A.A., M.H., H.M., M.K.; *Data Curation*, A.Z., A.A., A.A., M.H., H.M., L.A., H.E., W.A.; *Formal Analysis*, A.Z., A.A., A.A.; *Methodology*, A.Z., A.A., M.H., M.K.; *Project Administration*, A.Z., A.A., H.M., L.A., M.K.; *Resources*, A.Z., M.H.; *Supervision*, A.Z.; *Validation*, A.Z.; *Writing‐Original Draft*, A.Z., A.A., A.A., M.H., H.M., L.A., H.E., M.K., W.A.; *Writing‐Review & Editing*, A.Z., A.A., A.A., M.H., H.M., L.A., H.E., M.K., W.A.; *Investigation*, A.A., M.H., H.M., L.A., H.E.

## ETHICAL STATEMENT

Ethical approval has been secured from the research ethics committee at the National Cancer Institute in Misurata. The approval allowed access to patients' name, age, sex, city, phone number, and imaging and pathology reports. Access to patients' records is granted without prior patient consent, as the study is considered a population‐based study and a form of cancer registry. According to the European Network of Cancer Registries' confidentially and ethics guidelines, it is not possible nor practical to request consent from all patients prior to being investigated with imaging or tissue sampling. The guidelines argue, conceivably, that the European Directive on Data Protection is non‐applicable to population‐based cancer registries, and they are, therefore, exempt from requiring explicit consent. Funding, despite several attempts at the city and national levels, no funding was secured to complete the study.

## Data Availability

The data that support the findings of this study are available from the corresponding author upon reasonable request.
